# Iron-Nicarbazin derived platinum group metal-free electrocatalyst in scalable-size air-breathing cathodes for microbial fuel cells

**DOI:** 10.1016/j.electacta.2018.04.190

**Published:** 2018-07-01

**Authors:** Benjamin Erable, Manon Oliot, Rémy Lacroix, Alain Bergel, Alexey Serov, Mounika Kodali, Carlo Santoro, Plamen Atanassov

**Affiliations:** a Laboratoire de Génie Chimique, Université de Toulouse, CNRS, INP, UPS, Toulouse, France; b 6T-MIC Ingénieries, 9 rue du développement, 31320, Castanet-Tolosan, France; c Department of Chemical and Biological Engineering, Center for Micro-Engineered Materials (CMEM), Advanced Materials Lab, 1001 University Blvd. SE Suite 103, MSC 04 2790, University of New Mexico Albuquerque, NM, 87131, USA

**Keywords:** Cathode, PGM-Free catalysts, Oxygen reduction reaction, Microbial fuel cell, Power generation

## Abstract

In this work, a platinum group metal-free (PGM-free) catalyst based on iron as transitional metal and Nicarbazin (NCB) as low cost organic precursor was synthesized using Sacrificial Support Method (SSM). The catalyst was then incorporated into a large area air-breathing cathode fabricated by pressing with a large diameter pellet die. The electrochemical tests in abiotic conditions revealed that after a couple of weeks of successful operation, the electrode experienced drop in performances in reason of electrolyte leakage, which was not an issue with the smaller electrodes. A decrease in the hydrophobic properties over time and a consequent cathode flooding was suspected to be the cause. On the other side, in the present work, for the first time, it was demonstrated the proof of principle and provided initial guidance for manufacturing MFC electrodes with large geometric areas. The tests in MFCs showed a maximum power density of 1.85 W m^−2^. The MFCs performances due to the addition of Fe-NCB were much higher compared to the iron-free material. A numerical model using Nernst-Monod and Butler-Volmer equations were used to predict the effect of electrolyte solution conductivity and distance anode-cathode on the overall MFC power output. Considering the existing conditions, the higher overall power predicted was 3.6 mW at 22.2 S m^−1^ and at inter-electrode distance of 1 cm.

## Highlights

• Fe-based material was used as catalyst for ORR in MFCs.
• The catalyst was incorporated into a scalable-size air breathing cathode.
• MFC had an initial power density of 1.85 W m^−2^.
• The effect of solution conductivity and electrodes distance is evaluated on MFC performance.
• Higher power predicted was 3.6 mW at 22.2 S m^−1^ and at electrode distance of 1 cm.

## Graphical abstract

Iron-Nicarbazin derived platinum group metal-free electrocatalyst was incorporated into a scalable-size air-breathing cathodes and tested in microbial fuel cells. Maximum power density of 1.85 W m^−2^ was achieved initially and then decreased to 1.25 W m^−2^ after 22 days operations.

**Figure:**
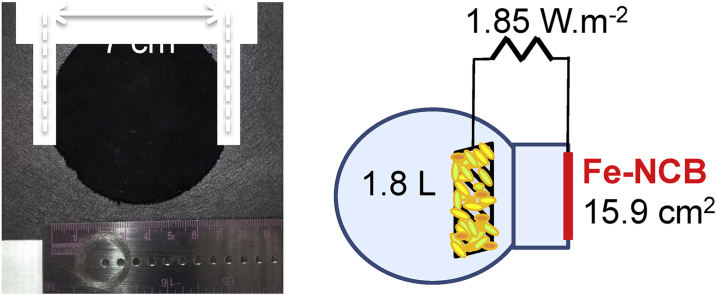

## 1 Introduction

Bioelectrochemical systems (BES) are relatively new technologies able to use biotic anodic reaction for degrading organics and cathodic biotic/abiotic reactions, naturally occurring or supported by an external power device, for generating electricity or producing value added products (VAPs) [[1], [2], [3], [4], [5], [6]].

BESs are cathodic-limited and consequently part of the ongoing research is focused on the reduction side of the overall red-ox reaction [[7], [8], [9], [10], [11], [12]]. Limitations are mainly due to the low kinetics of oxygen reduction (ORR) caused by room temperature operations and circumneutral pH electrolyte and high activation overpotentials [10,11]. Consequently, a catalyst is needed for increasing the ORR kinetics at the cathode.

By far, platinum has been the most utilized as catalysts for oxygen reduction reaction in operating microbial fuel cells (MFC) [[13], [14], [15]]. Lately, Pt or platinum group metal (PGM) catalysts are just used for comparison with novel and low cost cathodic materials [15]. This option has been abandoned due to platinum high cost (and low power generated) as well as low durability in presence of anions [[16], [17], [18], [19]], especially sulfur [20].

In order to mitigate those disadvantages there are two options were explored. The first is based on utilization of high surface area and high conductivity carbonaceous materials as ORR catalysts [[21], [22], [23], [24], [25], [26], [27]]. The second one proposes the usage of platinum group metals free (PGM-free) catalysts based on M-N-C active centers in which M is a transition metal such as Fe, Co, Ni, Mn and Cu [14,28,29]. Activated carbon (AC) as catalyst on a metallic current collector for MFC was firstly reported in 2009 by Zhang et al. [30]. Since then, it is by far the most adopted carbonaceous-based catalyst used for ORR in MFCs [14]. The concurrent low cost, commercial availability in large quantity, relatively high performances due to the high surface area and high chemical and mechanical stability in long time operation makes it a suitable candidate for MFCs applications [[31], [32], [33], [34]]. Also other conductive and high surface area carbonaceous materials named carbon nanotubes (CNTs) [35], carbon nanofibers (CNFs) [36], modified carbon black (CB) [37,38], graphene [[39], [40], [41], [42]] and others have been successfully utilized as cathode in operating MFCs.

Still performances can be further increased since the activation overpotentials remained still high and quantified in roughly 400 mV [[7], [8], [9]]. In order to overcome those tedious initial thermodynamic losses, PGM-free catalysts have to be used. In fact, the open circuit potential (OCP) translates towards more positive values. Unfortunately, the activation overpotentials remained still high and measured in roughly 300 mV but lower compared to carbonaceous catalysts [[43], [44], [45], [46]]. Several examples have been showed in literature concerning the utilization of earth abundant transition metal such as Fe [[46], [47], [48], [49], [50], [51], [52], [53], [54], [55], [56]], Mn [[57], [58], [59]], Co [[60], [61], [62]], Cu [63,64] and Ni [65,66] as catalysts incorporated into cathode adopted in single chamber MFC. So far, Fe-N-C class of materials seems to be the most promising and performing catalysts among the above mentioned [17,18,47,50,55,56]. Moreover, Fe is also the most abundant, cheap and probably the more environmentally friendly among the earth abundant metals and consequently the most suitable for large scale applications in MFC. Among the presented literature, the catalyst is incorporated into air breathing cathode with relatively low dimension (area 3–7 cm^2^) [17,18,43,44,[47], [48], [49], [50], [51], [52], [53], [54], [55], [56]]. Scalability of those cathodes for larger reactors are needed and, to the best of our knowledge, not yet presented in literature.

In this study, iron based catalysts (Fe-NCB) have been integrated into MFCs working cathode operated in neutral media. This catalyst was synthesized using sacrificial support method (SSM). SSM is a technique that uses silica as support during the pyrolysis and then silica is washed out through etching. SSM is used to create the necessary morphology facilitating the accessibility of the active sites to the oxygen molecules and the reaction products removal. SSM was also used to synthesize other catalysts previously presented and studied in neutral media but in diverse operating conditions [67,68]. For the first time, the catalyst was incorporated into air-breathing cathode with diameter of 7 cm (area 39 cm^2^) and adapted to the used reactor with a real 15.9 cm^2^ exposed to the electrolyte. Innovatively, an air-breathing cathode of this dimension is proposed for larger scale applications. Electrochemical performances over 22 days are here presented. A numerical method was also used to describe the MFC performances.

## 2 Experimental section

### 2.1 Catalysts preparation

PGM-free materials were synthesized by UNM developed Sacrificial Support Method (SSM) [[69], [70], [71]]. A dispersion of Nicarbazin (NCB) as organic precursors rich in nitrogen dispersed in water was deposited on the surface of two silica: OX-50 (surface area ∼50 m^2^g^-1^) and in-house synthesized monodispersed silica (surface area ∼10 m^2^g^-1^, particle size ∼50 nm). The suspension of silica and organic precursor was mixed together under ultrasonication followed by addition Fe(NO_3_)_3_*9H_2_O. The mass ratio between of iron nitrate and organic precursor was selected as 1:8. Water was evaporated on air at T = 85 °C. The dry composite mixture was ground with mortar and pestle till a fine powder was achieved. The fine powder was subject to heat treatment in inert atmosphere (ultra high purity (UHP) N_2_ flow rate of 100 ccm) at T = 975 °C for 45 min. The final temperature of 975 °C was reached with a ramp rate of 10°Cmin^-1^. After 45 min, the fine powder was cooled down at room temperature. Silica was removed by means of 25 wt% of HF and duration of leaching was 24 h. The catalyst was washed using DI water until neutral pH and dried at T = 85 °C for ∼12 h. Obtained powder was subject to an additional heat treatment in NH_3_ atmosphere (flow rate of 100ccm) at T = 975 °C for 30 min. The second heat treatment was shown to be beneficial for the catalyst performances as previously reported [67].

### 2.2 Air breathing cathode preparation

**Fig. 1:**
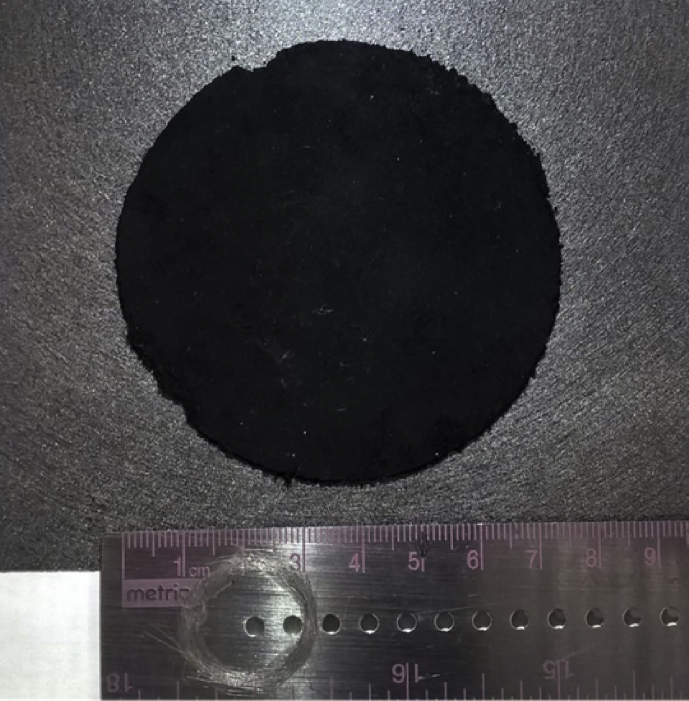
Image of the air-breathing cathode.

The air-breathing cathode used in this investigation was a pellet-type cathode with a mixture of powder and binder pressed over a carbon paper used as current collector. Particularly, a blender was used for mixing 70 wt% activated carbon (AC, SX Ultra, Sigma Aldrich), 10% carbon black (CB, Alfa Aesar, 50% acetylene), and 20 wt% PTFE (60% emulsion, Sigma Aldrich) for 5 min continuously. The mix was inserted into a metallic pellet die (diameter 7 cm) and pressed at 2 mT for 5 min using a Carver hydraulic press. The AC cathode was used as control. In the case of Fe-based cathode, Fe-based catalyst was mixed within the AC/CB/PTFE mixture and then pressed over the current collector. Carbon paper (Spectracarb™, 2050A-2050, Engineered Fiber Technology LLC) was used as current collector. The cathode image was here reported (Fig. 1). AC/CB/PTFE loading was 30 mg cm^−2^ and the catalyst loading was 1 mg cm^−2^. AC/CB/PTFE based cathode (30 mg cm^−2^ loading) without the addition of Fe-NCB catalyst was also tested as control. The cathode surface area was ≈39 cm^2^ but it was adapted to the reactor used during the experiments and the area exposed to the electrolyte was 15.9 cm^−2^.

### 2.3 Surface morphology

Scanning Electron Microscopy (SEM) Hitachi S-800 instrument was used to image the catalyst surface to determine morphological characteristics.

### 2.4 Anodic biofilm formation under chronoamperometry

The formation of biofilm on anode was carried out directly in a removable air-cathode MFC reactor design first introduced by Oliot et al. [72]. The reactor had a volume of 1.8 L. The anodic biofilm was formed from compost leachate with 20 mM acetate as the substrate using a 3-electrode electrochemical set-up. A 40 cm^2^ carbon cloth electrode (PaxiTech, France) was used as working electrode for supporting biofilm. A platinum grid was used as the auxiliary electrode and a saturated calomel electrode (SCE) as the reference (+0.242 V/SHE). The working electrode was constantly polarized at −0.2 V/SCE using a MPG2 potentiostat (Bio-Logic SA, France) during the biofilm formation. All experiments were carried out at 40 °C in a thermo regulated oven. Once mature biofilm were producing beyond 10 A m^−2^, the compost leachate was changed by a fresh synthetic electrolyte (50 mM bicarbonate buffer supplemented by a macronutrients solution 10 mL L^−1^, a micronutrients solution 1 mL L^−1^, vitamins 1 mL L^−1^, KCl 4.5 g L^−1^ and NaH_2_PO_4_·H_2_O 2.4 g L^−1^; pH adjusted to 7.0) and the microbial anode were polarized for 10 additional days in order to achieve a steady current generation regime (under a constant concentration of 20 mM acetate).

### 2.5 MFC operation

The Fe-NCB air-breathing cathode was assembled to the removable air-cathode MFC with a stainless steel current collector maintained by a PVC screw cap designed with a central hole of 4.5 cm diameter (all the details are given in Oliot et al. [72]). The surface area of the air-breathing cathode exposed to the electrolyte was 15.9 cm^2^. During MFC operation, acetate concentration was maintained at 20 mM by daily measurements and adjustments. Power/current curves were recorded periodically using a variable external resistance ranging from 1 Ω to 33 kΩ. A high-impedance voltmeter (Keithley, 2000 multimeter, USA) measured the cell voltage and a second voltmeter measured the anode and cathode potentials versus the SCE reference. MFCs produced power continuously through a 33 Ω electrical resistance. Power and current densities were calculated relative to the 15.9 cm^2^ air-cathode surface area.

### 2.6 Numerical modelling of MFC performance

The numerical model was based on the secondary distribution of the electrostatic potential inside the fuel cell, described by the Laplace equation. The theoretical basis and the numerical procedure have been detailed previously for the case of a microbial electrolysis cell [73] and of a MFC [74,75]. Secondary current distribution was considered, using the electrochemistry module of the COMSOL Multiphysics^®^ software. The definition of the geometry of the reactor was carefully based on the MFC design experimentally used. The Laplace equation solved in the electrolyte domain:

(1) $$\Delta \varphi_{s}=0$$

leads to the field of electrostatic potential and then to the local current by using Ohm's law:

(2) $$i_{s}=-\sigma_{s}\nabla \varphi_{s}$$

with σ_S_: liquid phase conductivity (S m^−1^), φ_S_: electrolyte potential (V) and i_S_: electrolyte current density (A m^−2^).

A Nernst-Monod equation was used as input for the kinetic of the anodic reaction at the electrode/electrolyte interface:

(3) $$J=\frac{J_{max}}{1+exp\left(\frac{-nF\left(E-E_{\frac{1}{2}}\right)}{RT}\right)}$$

with J_max_: maximum current density (A m^−2^), n: number of electrons involved in the reaction (dimensionless); F = 96 500C mol^−1^; R: 8.314 J mol^-1^ K^-1^; E: electrode potential (V) and E_1/2_: electrode potential value corresponding to the half the J_max_ (V).

A Butler-Volmer equation was used as input for the kinetic of the cathodic reaction at the electrode/electrolyte interface:

(4) $$J=J_{0}\left[exp\left(\frac{-\alpha_{a}\;n\;F\;\eta }{R\;T}\right)-exp\left(\frac{-\alpha_{c}\;n\;F\;\eta }{R\;T}\right)\right]$$

with J_0_: exchange current density (A m^−2^), α_a_: anodic charge transfer coefficient (dimensionless), n: number of electrons involved in the reaction (dimensionless), F: 96 500C mol^−1^, η: overpotential (V); R: 8.314 J mol^-1^ K^-1^, α_c_: cathodic charge transfer coefficient (dimensionless).

Solid phase potential were considered homogeneous within the electrodes due to the non-limited electrical conductivity of carbon materials. A similar approach was already considered in previous works published [[73], [74], [75]]. Theoretical MFC performance modelling was performed varying (i) inter-electrodes distances (distance between the centers of each electrode) ranging from 10 to 3.5 cm with steps of 0.5 cm; and (ii) the conductivity of the electrolyte. Four increasing values of electrolyte ionic conductivity, corresponding to more and more saline environments, were considered (Table 1).

**Table 1:** Values of the liquid phase conductivity considered in this study.

Electrolyte	Ionic conductivity, S.m^−1^
Synthetic medium (40 °C)	1.25 (experimentally measured)
Compost leachate (40 °C)	0.88 (experimentally measured)
Seawater (20 °C)	5.30 [75]
25% (w/w) NaCl solution^a^ (20 °C)	22.20 [76]

a Minimum salinity of salt lakes.

## 3 Results and discussion

### 3.1 Catalyst surface characteristics

**Fig. 2:**
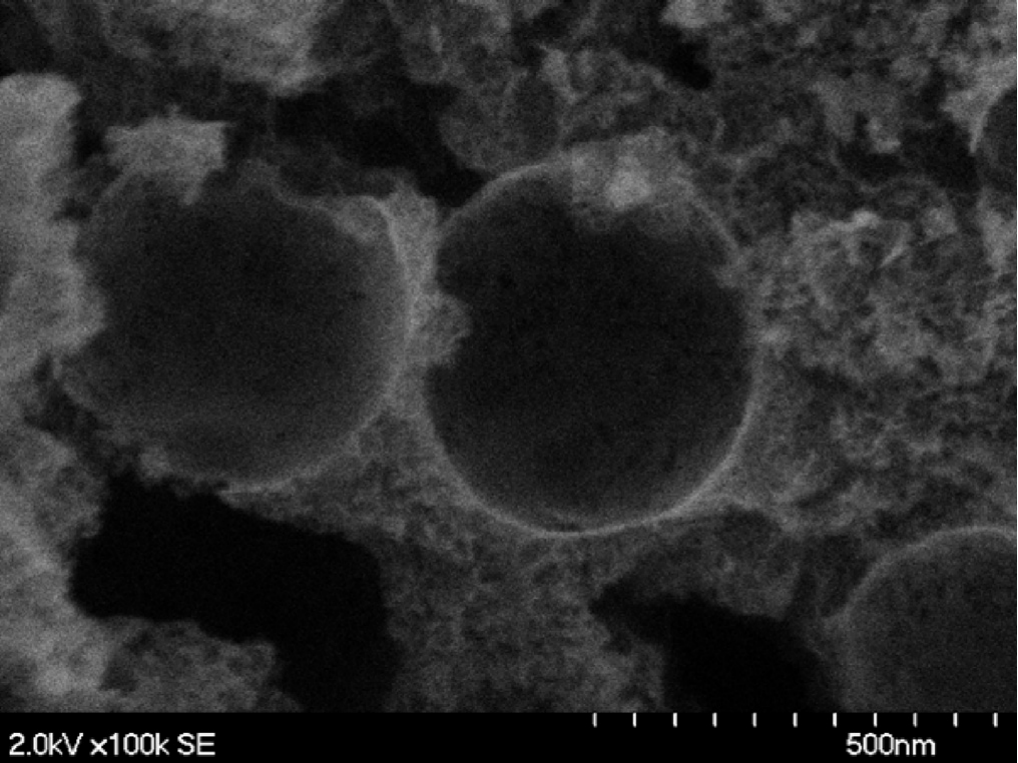
SEM image of Fe-NCB catalyst prepared by Sacrificial Support Method.

Catalyst morphology was imaged by SEM and presented on Fig. 2. It can be clearly seen that material consist of two different sets of pores: i) large pores formed after leaching of ∼50 nm monodispersed silica and ii) smaller pores which were created during pyrolysis of organic precursor material. The overall BET surface area of catalyst was ≈560 m^2^ g^−1^. Such morphology of M-N-C electrocatalysts synthesized by Sacrificial Support Method (SSM) was previously reported the details were explained in published literature [[77], [78], [79]].

### 3.2 Cathodic ORR performance in MFC

The electro-catalytic activity of Fe-NCB was previously discusses through rotating ring disk electrode (RRDE) experiments in oxygen saturated neutral media [68]. In fact, Fe-NCB had higher catalytic activity compared to platinum and AC [68]. Particularly, Fe-NCB had higher half-wave potential and lower peroxide production indicating a more efficient ORR [68]. The peroxide yield produced by Fe-NCB was lower than 10% while the one from AC was between 30% and 60% [68]. It is well known that AC and carbonaceous catalyst follow a 2e-transfer mechanism during ORR with production of the only reaction intermediate. In parallel, it was shown that Fe-NCB and Fe-based catalyst follow a 2x2e-transfer mechanism with the intermediate formed that is further reduced on another active center [47,50,55,67,68].

**Fig. 3:**
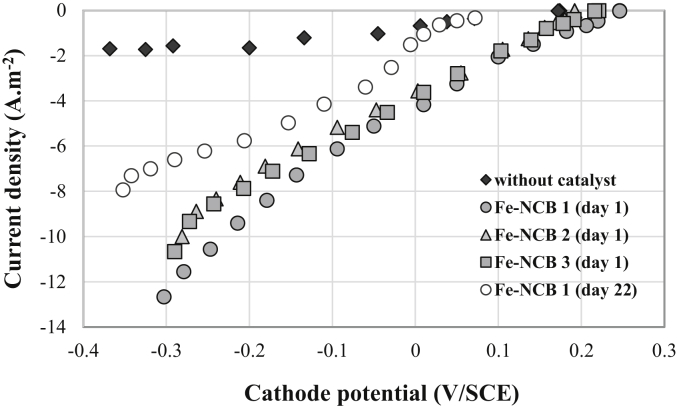
Comparison of cathode kinetics (I-E curves) measured during MFC polarization with three different Fe-NCB air-breathing cathodes and with an air-breathing cathode without catalyst.

After the air-breathing cathodes containing or not Fe-NCB catalyst were installed for a 24 h period on the MFC single-chamber reactor, polarization curves were run for characterizing the electro-catalytic behavior of the cathodes with respect to the oxygen reduction reaction (Fig. 3, day 1). The open-circuit potential (OCP) of the air-breathing cathodes loaded with Fe-NCB catalyst (Fe-NCB 1, Fe-NCB 2, Fe-NCB 3) was close to +0.25 V/SCE. The same electrode without catalyst had a free potential of +0.18 V/SCE after 1 day of exposure to the synthetic electrolyte. The addition of Fe-NCB produced an advantage of roughly +0.07 V on the OCP. Those results are in good agreement with previously reported data on PGM-free catalysts for MFCs [17,18]. The theoretical potential value for ORR in neutral media (pH 7) is +0.57 V/SCE and therefore the value obtained in this experimentation indicated activation overpotentials of 0.32 V. Such high losses values are in agreement with previously reported literature [17,18].

The catalytic effect associated with the presence of Fe-NCB was remarkable since the cathode kinetics of the electro-reduction reaction of oxygen was greatly improved between a cathode loaded and not loaded with Fe-NCB (Fig. 3). Specifically, at a cathode potential of −0.2 V/SCE, the steady state cathodic current density is on average 8.0 A m^−2^ on an electrode containing the Fe-NCB catalyst, while the current density was less than 2.0 A m^−2^ on an iron-free electrode, at this same cathode potential. In addition, the electrocatalytic behavior with respect to the oxygen reduction reaction of the three cathodes loaded with Fe-NCB catalyst was relatively similar since the I-E curves, obtained experimentally with the three different cathodes, are almost superimposable as shown in Fig. 3.

In sum, both the catalytic activity and the experimental procedures for the manufacturing of the air-breathing cathodes and the deposition of the Fe-NCB catalyst seem all reproducible. AC is lately the most used cathode catalyst for its specific characteristics such as low cost, high performance and high durability in polluted environments [23,80]. The electrochemical performance here shown in neutral media underlined the advantage that is reached while using Fe-contained catalyst compared to simple AC. As the cost of Fe-NCB is generally considered low since both precursor (Nicarbazin) and iron salts are cheap and readily available, PGM-free utilization can be certainly considered as integration and upgrade to the most used AC based cathodes.

**Fig. 4:**
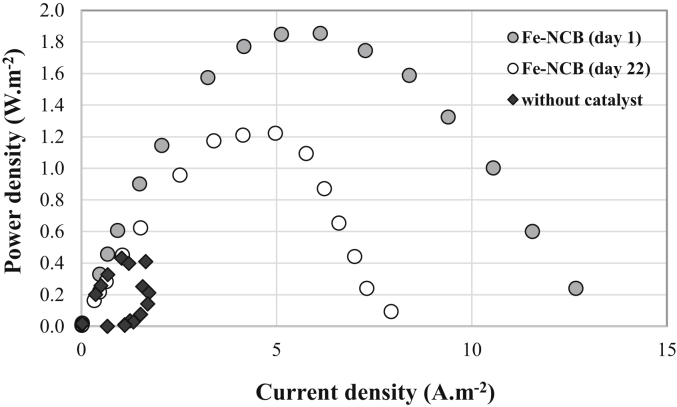
Polarization curves (P-I curves) of the MFC carried out at t_0_ with a cathode without catalyst and another containing Fe-NCB catalyst, and then after 22 days of operation.

**Fig. 5:**
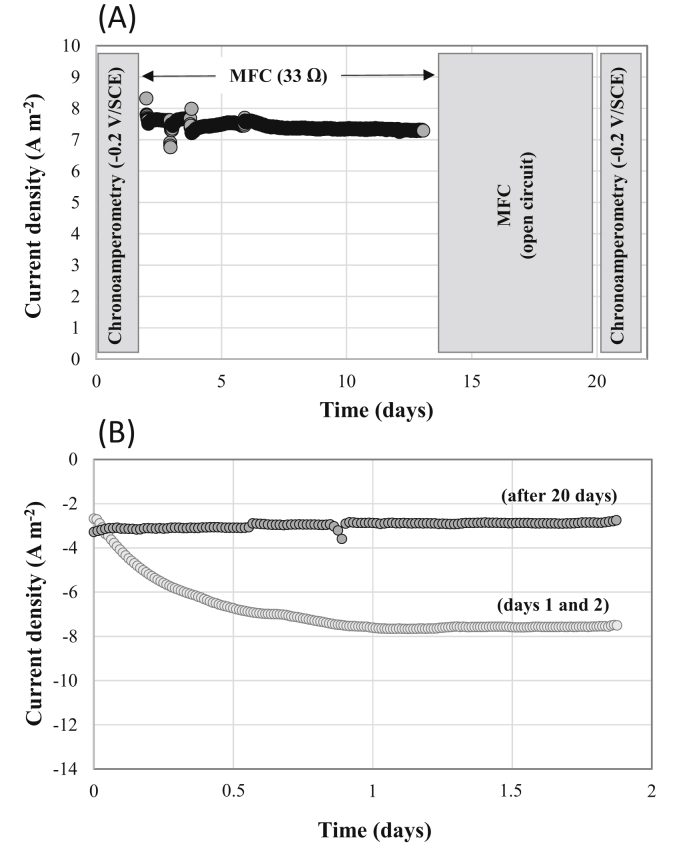
Current density delivered by the single chamber MFC with an external resistance of 33Ω between the bioanode and the Fe-NCB air-breathing cathode (A) and when the cathode was polarized for a 2-days period at −0.2 V/SCE (B).

In parallel, the anodic biofilm was first formed at −0.2 V/SCE on a carbon cloth electrode and stabilized in a synthetic electrolyte at 1.25 S m^−1^ of ionic conductivity, i.e. generating stable current at more than 10 A m^−2^. The use of the Fe-NCB catalyst on the cathodes greatly affected the overall performance of the single chamber MFC, both in terms of maximum current density and power density generated (Fig. 4). Thus, the maximum current density increased from 1.73 to 13.10 A m^−2^ (7.5 fold) and the maximum power density from 0.43 to 1.85 W m^−2^ (4.3 fold) over short measurement periods of 30 min when the cathode without catalyst is replaced by a cathode charged with Fe-NCB catalyst. For 12 days, the MFC measured a stable current density between 7.13 and 7.82 A m^−2^, through a constant load of 33 Ω (Fig. 5a). This resistance was specifically chosen because it corresponded to the resistance for which the generated power density was the maximum.

The MFC was then left in open circuit voltage for 6 days and Fe-NCB air-breathing cathode, which had been in contact with the electrolyte for a total of 20 days, was then polarized at −0.2 V/SCE for 2 days chronoamperometric test (Fig. 5a). The comparison of the 48 h chronoamperometry profiles obtained with the same Fe-NCB air-breathing cathode between day 0 and day 2 and then between day 20 and day 22 clearly showed a loss of catalytic activity. Indeed, the stationary current density at −0.2 V/SCE decreased from close to 8.0 A m^−2^ on day 2 to less than 3.0 A m^−2^ on day 22 (i.e. more than 62% of activity loss) (Fig. 5b). The drop in the cathode performance is even more apparent on the I-E curve of Fig. 3 (Fe-NCB1, day 22) on which it can be seen that both the open circuit potential of the cathode shifted to a 175 mV more negative value and the kinetics of oxygen electro-reduction has been slowed down beyond a current density of 6.0 A m^−2^ (the slope of the I-E curve was lower).

A change in the oxygen concentration ([O2] _t=22d_ < [O2] _t=2d_) and in the oxygen availability (oxygen transfer) in the vicinity of the Fe-NCB catalytic layer could explain first the decrease of the free potential of the cathode experimentally observed and then in another measure the limitation of the transfer of cathodic charge highlighted above a current density of 6.0 A m^−2^. This decrease in the oxygen concentration can have its origin in: i) the formation of an aerobic/anaerobic microbial biofilm on the cathode which can consume a large part of the oxygen which is exchanged in the cathode at the interface between the air and the electrolyte [[81], [82], [83]]; ii) the progression of the liquid electrolyte front by percolation inside the electrode which floods the electrocatalytic layer of Fe-NCB [84]. The increase of the pH close to the cathode [74,85,86], linked to the consumption of H^+^ and/or the production of OH^−^ ions during the oxygen reduction reaction at neutral pH following the alkaline pathway (O_2_ + 4e^−^ + 2H_2_O → 4 OH^−^), can also thermodynamically explain the shift of the cathode open circuit potential to more negative values.

This loss of cathode catalytic activity obviously had negative repercussions on the performance of the MFC evaluated on day 22 (Fig. 4, Fe-NCB day 22), with respective reductions of 35% of the maximum power densities (P_max_) and the maximum current densities (J_max_) supplied by the MFC (Fig. 4).

### 3.3 Understanding cathodic performance loss

Basically, the decrease in the performance of air-breathing cathodes used in MFCs is generally related to (i) deactivation or pollution of the abiotic catalyst [[16], [17], [18]], (ii) fouling of the cathode by an organic deposit (often a microbial biofilm) or inorganic precipitates associated with a local pH increase [43,81,84,87], (iii) a pH change thermodynamically unfavorable to the oxygen reduction reaction [85,86], (iv) wetting/flooding of the cathode due to percolation of the electrolyte through the cathode or poor management of the produced water or condensation of water [84].

Here, the intrinsic catalytic activity of the Fe-NCB catalyst does not seem to have been really impacted since the electrochemical kinetics for current densities less than 6.0 A m^−2^ have not been significantly modified (Fig. 3). Consequently, the hypotheses concerning the possible deactivation, as well as either biological or inorganic pollution of the catalyst appear to be reasonably discarded. It was recently showed a slight decrease in performance using rotating ring disk electrode (RRDE) technique over 10 000 cycles on similar Fe-contained catalyst in neutral media [88]. At last, it was shown that Fe-N-C catalysts are very stable in environments containing anions such as S^2−^, SO_4_^2−^ and Cl^−^ generally responsible for Pt deactivation [[16], [17], [18], [19]]. The hypothesis of a significant change in pH near the cathode is also unlikely since the experimental system of MFC used did not involve an ionic membrane.

Only the formation of a microbial biofilm on the cathode, strongly constraining the mobility of the OH^−^ ions produced on the cathode, could induce a strong alkalization of the microenvironment close to the electrocatalytic surface leading to an important decrease in the cathode OCP as measured (Fig. 3).

On the other hand, several hypotheses could wholly or partially justify the significant decrease in the oxygen concentration close to the catalytic sites where the oxygen reduction reaction takes place. The first possible hypothesis could again concern the development of an aerobic/anaerobic microbial biofilm on the face of the cathode exposed to the electrolytic liquid medium. The layers of bacteria closest to the catalytic surface of the cathode would then locally consume oxygen as it was solubilized in the electrolyte [[81], [82], [83]]. Another possibility is dictated to the fact that the alkalization of the cathode favors the precipitation of carbonates and other inorganic salts that cover the catalytic active sites reducing the overall performance [82,87]. This layer of inorganic fouling might also act as barrier increasing the overall proton mass transfer resistance and lower the cathode performance [43,80,82,87]. The other possibility is that the cathode completely floods after a certain operating time due to the gradual losses of the cathode hydrophobic properties and the aqueous electrolyte gradually/suddenly pass through the cathode structure filling the empty pores. In this case, only dissolved oxygen which concentration is quite low, can be used as oxidant reagent.

**Fig. 6:**
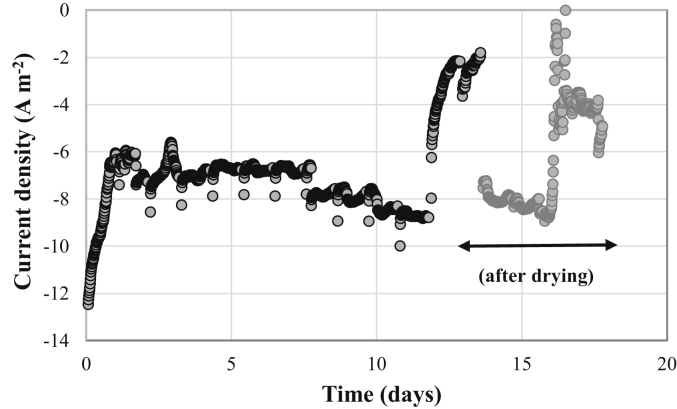
Current density measured on a Fe-NCB air-breathing cathode in contact with a “clean” 100 mM carbonate electrolyte at pH 7.8 with stirring.

A new test for the stability of Fe-NCB air-breathing cathode was carried out in a completely aseptic “clean” system, i.e. in contact with a clean electrolyte without any source of microorganisms (neither inoculum, nor bioanode) in order to avoid the phenomenon of biofouling of the cathode. The sterile aqueous electrolyte was buffered (100 mM carbonate, pH 7.8) and stirred to minimize any phenomenon of local pH variation on the cathode surface. Under these conditions, Fe-NCB air-breathing cathode, polarized at −0.2 V/SCE, generated a cathodic current of 6.0–8.0 A m^−2^ for 11 days after a first short rise period in steady state of 24 h (Fig. 6). Then suddenly, after 12 days, the reduction current suddenly collapsed at 2 A m^−2^ in less than 12 h. The visual appearance of the face of the cathode exposed to air showed signs of liquid water present between the current collector and the surface of the electrode. The cathode was then disassembled and then analyzed rapidly under an optical microscope and no particular biological or inorganic deposition was found on the surface of the cathode exposed to the electrolyte. In fact, as no bacteria were introduced, and no carbonate species were present into the buffer solution, biofilm attachment and inorganic fouling was not expected. The cathode was subjected to a drying treatment in an oven at 80 °C for 12 h and then re-positioned on the abiotic MFC reactor (used without any microorganism). Immediately, the cathode again produced a stable cathodic current of 8.0 A m^−2^ on average for 48 h. Then, on day 16, the electrolyte again succeeded in percolating through the Fe-NCB air-breathing cathode, resulting in a drop of more than 60% of the current density experimentally measured on the polarized cathode.

This sudden decrease in electrochemical performances might be due to gradual loss of the three phase interphases (TPI) and the consequent cathode flooding. While it seems that the air-breathing cathode structure as it is built was able to work properly for 12 days, the structure loose its hydrophobic/hydrophilic properties leading to flooding and actually complete “perforation” of the cathode structure with the solution leaking from the structure vigorously. The water dripping out from the cathode to the external environment indicates that the cathode is fully flooded and, the maximum oxygen concentration possible is only the maximum solubility of the oxygen in aqueous solution (9.07 mg L^−1^ or 0.28 mM at 40 °C.). This could explain the sudden and step drop in current density produced. It was also noticed that the cathode exposed directly to the solution was slowly dissolving into the electrolyte. Interestingly, the drying procedure can recover completely the performance and actually produce similar output but only for a short amount of time. This might be due to the fact that the salts within the electrolyte that were flooding the electrode could close temporarily the pores and stop the electrolyte from percolating outside. After the exposure to the electrolyte for additional two days, those salt precipitations might be re-dissolved leading to the creation of preferential pathways for the electrolyte to move externally.

### 3.4 Evaluation of the theoretical maximum MFC power output by electrochemical modelling

**Fig. 7:**
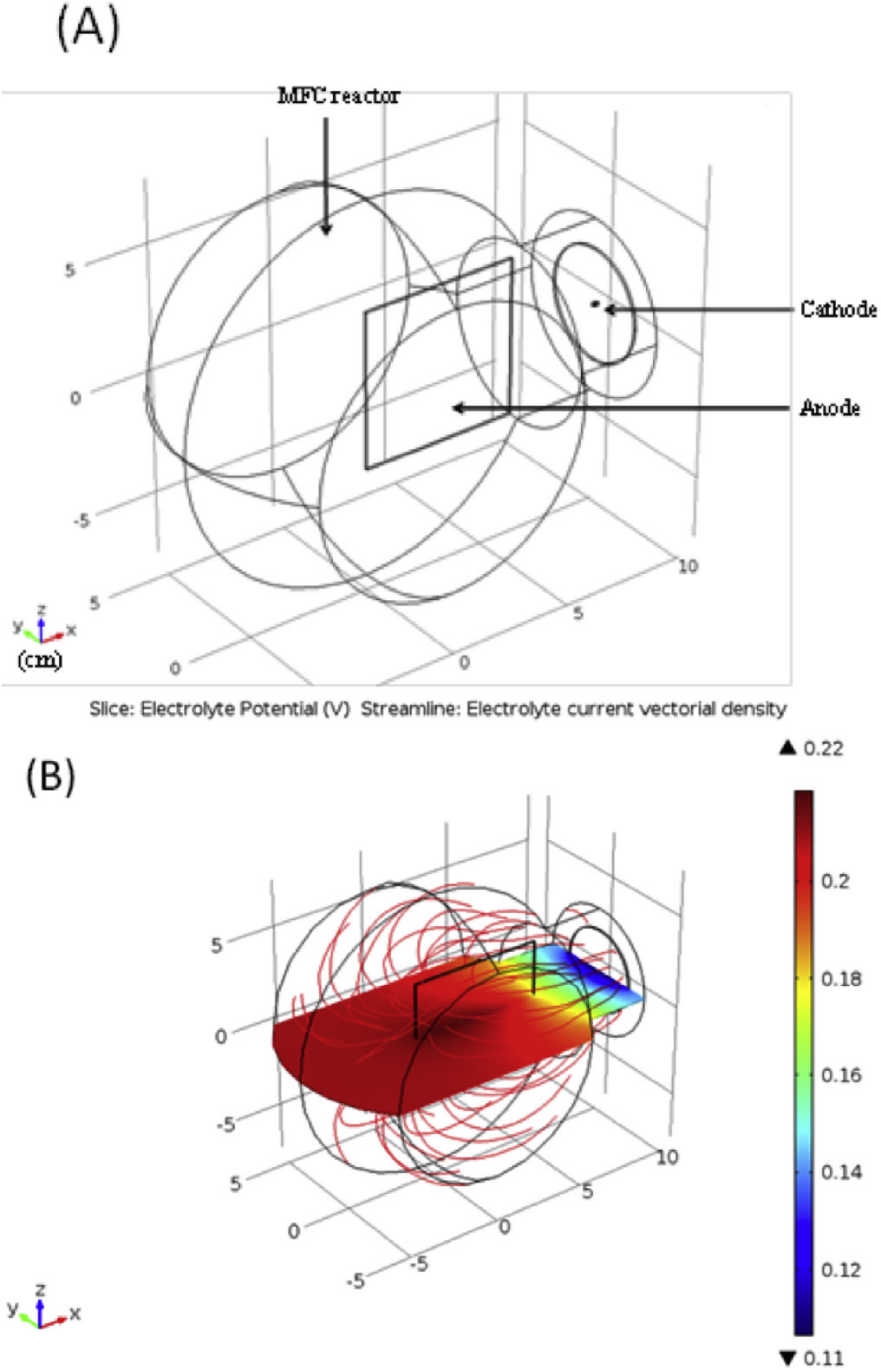
Calculation of the electrolyte potential distribution for a MFC power output of 2.12 mW.

The measurement of the cathodic potential during the polarization of the MFC (Fig. 3, Fe-NCB) as well as the linear sweep voltammetries carried out on the bioanodes made it possible to extract the individual (bio) electrochemical kinetics of the Fe-NCB air-breathing cathode and the bioanode respectively. From the obtained kinetics, a theoretical calculation to extrapolate the optimal achievable MFC performance was attempted by using increasingly conductive electrolytes as well as by reducing the working distance between the cathode and the bioanode. Fig. 7 presents the result of potential distribution calculation for an ionic conductivity value of 1.25 S m^−1^, a cell voltage of 0.3 V and a distance between the center of the anode and the center of the cathode of 7 cm. This voltage value is associated with the maximum power output (2.12 mW) by the specific design of MFC experimentally used in this study.

Relatively non-negligible potential gradient along the surface of the anode (around 50 mV), corresponding to ohmic drop in the liquid phase was evaluated. The corresponding effect was a higher current density at the anode/electrolyte interface in the areas located closer to the cathode (1.54 A m^−2^ for a distance of 3 cm) compared to the more distant area (0.77 A m^−2^ for a distance of 9 cm). Results are consistent with previous calculations already demonstrated [73,75].

**Fig. 8:**
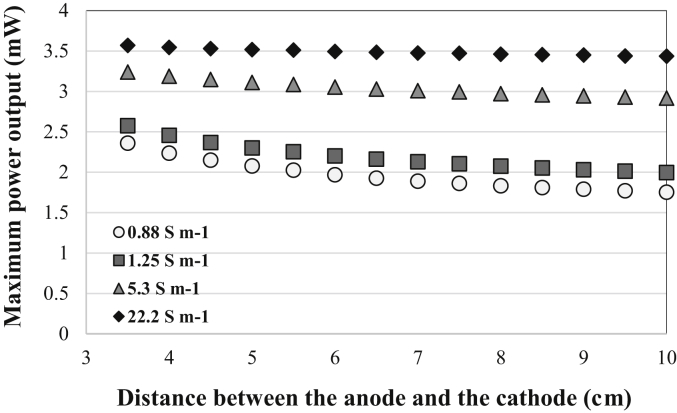
Influence of the ionic conductivity and the distance between the center of the bioanode and the center of the air-breathing cathode on the maximum MFC power output.

Fig. 8 details the result of the calculation of the maximum MFC power output (expressed in mW) as a function of the inter-electrode distance (between the center of the electrodes) for four increasing values of ionic conductivity corresponding to the conductivities of synthetic electrolyte, compost leachate, seawater and 25% NaCl solution (Table 1).

The influence of the geometry of the system (inter-electrode distance) strongly depends on the electrolyte ionic conductivity. When highly concentrated NaCl (25%) aqueous solution is used (22.2 S m^−1^), the MFC maximum power output can reach 3.6 mW and does not consequently depend on the inter-electrode distance. For lower ionic conductivity values (<10 S m^−1^), the power performance of the MFC is basically improved by decreasing the inter-electrode distance. The ratio of power output with minimum inter-electrode distance (compared to the configuration with an anode in the center of the reactor) equals 1.34 (+34%) for a conductivity of 0.88 S m^−1^; 1.29 (+29%) for a conductivity of 1.25 S m^−1^ and 1.11 (+11%) for a conductivity of 5.3 S m^−1^.

### 3.5 Outlook on future improvement of the catalyst layer stability

Cathode electrochemical performance are mainly responsible for the low power generation in bioelectrochemical system. In this work, air-breathing cathode fabricated mixing AC, CB, PTFE and Fe-NCB was tested in operating MFC. Differently than previously reported work, in this case, a tentative of enlarging the cathode area was pursued and the pellet die utilized was much larger with a nominal diameter of 7 cm compared to the usual adopted of ≈3 cm. The cathode obtained was then adapted to the existing single chamber MFC and only 15.9 cm^2^ was facing the electrolyte solution but still more than double than the electrode area generally utilized in this type of experiments. Scaling up of the cathode is also another important issue to face for practical implementations. Unfortunately, as larger the cathode area to fabricate becomes as more difficult the uniformity of the cathode area becomes. In fact, the electrolyte leakage was not uniform all over the external surface, but it was localized in specific points. Moreover, it was noticed that the cathode consistency tends to decrease over time, even in abiotic conditions, with the partial dissolution of the black powder into the electrolyte. A different hydraulic pressing strategy, an increase in the PTFE binder or automated powder dispersion on the current collector to guarantee uniform distribution might be a possible solution to further study and investigate.

In order to avoid this situation, several alternative solutions named: i) utilization of membrane, ii) utilization of external diffusion layers, iii) change in the cathode preparation, might be considered. Firstly, a membrane separator (e.g. ceramic [89,90], polymeric [91,92], etc) could be assembled with the cathode in a membrane-electrode assembly (MEA) configuration in order to avoid powder dissolution. Membrane is generally negatively identified as part of the MFC due to the high cost and the increase in the ohmic losses with a decrease in the electrochemical output [93]. Secondly, external diffusion layers such as PTFE [94], poly(dimethylsiloxane) (PDMS) [95], etc [96] could be applied to the external face of the cathode in order to prevent leakage. This solution was successfully adopted previously [31] but still, the addition of external layers is not beneficial for the oxygen diffusion from the atmosphere to the catalytic sites and therefore the performance might be penalized. Thirdly, it might be possible that the pellet of AC, CB, PTFE and Fe-NCB did not stick properly to the carbon paper substrate used as current collector. Future improvements should take into account a utilization of metallic meshes (e.g. stainless steel or nickel) as current collectors and different mesh size should be tested till an optimum is found. An increase in the PTFE binder percentage within the cathode mixture should be also pursued.

Considering the electrochemical performance of the cathodes investigated with and without additional Fe-NCB catalyst, it was found that the Fe-based catalyst gave a significant boost in the performance compared to bare and untreated commercial AC. As the cost of Fe-based catalyst is generally low, PGM-free catalyst should substitute the utilization of simple and commercial AC and be finally considered as the baseline for future MFCs improvements. Maximum power density achieved by the Fe-NCB cathode initially was 1.85 W m^−2^ (day 1) and then decrease by roughly 30% at 1.25 W m^−2^ after 22 days operations. The maximum power density here reported is comparable to the performance achieved by iron-based catalysts synthesized using the polymerization-pyrolysis method and a catalyst loading of 1 mgcm^−2^ [[97], [98], [99]]. The performance are instead slightly lower compared to other reported literature in which the peak of current density was above 2 Wm^-2^ when Fe-based catalyst was incorporated into the air-breathing cathode structure [17,18,43,44,47,50,56,[67], [68], [69]]. Nevertheless, in this experimentation compared to literature, low electrolyte solution conductivity (1.25 S m^−1^), low catalyst loading (1 mg cm^−2^) and larger cathode area (15.9 cm^2^) was tested. The performances might increase if electrolyte solution conductivity is increased as well as if also the distance between anode and cathode is reduced as shown by the numerical model.

## 4 Conclusions

Iron-Nicarbazin (Fe-NCB) was prepared using SSM and tested as PGM-free catalyst into MFCs systems. The catalyst was merged into the cathode structure and tested in abiotic conditions and in operating MFCs. After several days of tests, in both abiotic and biotic operating conditions, the cathode lost its hydrophobic properties leaking through the external environment. This might be due to the not uniformity in fabricating larger scale cathode. Further improvements should be studied and pursued. Maximum power density of 1.85 W m^−2^ was achieved initially and then decreased to 1.25 W m^−2^ after 22 days operations. Fe-NCB had much higher performance compared to Fe-free catalyst with only AC acting as catalyst. The numerical model aiming to predict the effect of electrolyte solution conductivity and distance anode-cathode on the overall MFC power output showed that the highest power predicted was 3.6 mW at 22.2 S m^−1^ and at inter-electrode distance of 1 cm.

## References

[1] Rinaldi A., Mecheri B., Garavaglia V., Licoccia S., Di Nardo P., Traversa E. (2008). Engineering materials and biology to boost performance of microbial fuel cells: a critical review. Energy Environ. Sci..

[2] Santoro C., Arbizzani C., Erable B., Ieropoulos I. (2017). Microbial fuel cells: from fundamentals to applications. A review. J. Power Sources.

[3] Pandey P., Shinde V.N., Deopurkar R.L., Kale S.P., Patil S.A., Pant D. (2016). Recent advances in the use of different substrates in microbial fuel cells toward wastewater treatment and simultaneous energy recovery. Appl. Energy.

[4] Bajracharya S., Srikanth S., Mohanakrishna G., Zacharia R., Strik D.P., Pant D. (2017). Biotransformation of carbon dioxide in bioelectrochemical systems: state of the art and future prospects. J. Power Sources.

[5] Brown R.K., Schmidt U.C., Harnisch F., Schröder U. (2017). Combining hydrogen evolution and corrosion data - a case study on the economic viability of selected metal cathodes in microbial electrolysis cells. J. Power Sources.

[6] Guo K., Prévoteau A., Rabaey K. (2017). A novel tubular microbial electrolysis cell for high rate hydrogen production. J. Power Sources.

[7] Rismani-Yazdi H., Carver S.M., Christy A.D., Tuovinen O.H. (2008). Cathodic limitations in microbial fuel cells: an overview. J. Power Sources.

[8] Madjarov J., Popat S.C., Erben J., Gçtze A., Zengerle R., Kerzenmacher S. (2017). Revisiting methods to characterize bioelectrochemical systems: the influence of uncompensated resistance (iRu-drop), double layer capacitance, and junction potential. J. Power Sources.

[9] Erable B., Feron D., Bergel A. (2012). Microbial catalysis of the oxygen reduction reaction for microbial fuel cells: a review. ChemSusChem.

[10] Kinoshita K. (1988). Carbon: Electrochemical and Physicochemical Properties.

[11] Kinoshita K. (1992). Electrochemical Oxygen Technology.

[12] Ucar D., Zhang Y., Angelidaki I. (2017). An overview of electron acceptors in microbial fuel cells. Front. Microbiol..

[13] Wang Z., Cao C., Zheng Y., Chen S., Zhao F. (2014). Abiotic oxygen reduction reaction catalysts used in microbial fuel cells. ChemElectroChem.

[14] Wang Z., Mahadevan G.D., Wu Y., Zhao F. (2017). Progress of air-breathing cathode in microbial fuel cells. J. Power Sources.

[15] Yang W., Kim K.-Y., Saikaly P.E., Logan B.E. (2017). The impact of new cathode materials relative to baseline performance of microbial fuel cells all with the same architecture and solution chemistry,. Energy Environ. Sci..

[16] Tylus U., Jia Q., Hafiz H., Allen R.J., Barbiellini B., Bansil A., Mukerjee S. (2016). Engendering anion immunity in oxygen consuming cathodes based on Fe-N x electrocatalysts: spectroscopic and electrochemical advanced characterizations. Appl. Catal., B.

[17] Santoro C., Serov A., Narvaez Villarrubia C.W., Stariha S., Babanova S., Artyushkova K., Schuler A.J., Atanassov P. (2015). High catalytic activity and pollutants resistivity using Fe-AAPyr cathode catalyst for microbial fuel cell application. Sci. Rep..

[18] Santoro C., Serov A., Stariha L., Kodali M., Gordon J., Babanova S., Bretschger O., Artyushkova K., Atanassov P. (2016). Iron based catalysts from novel low-cost organic precursors for enhanced oxygen reduction reaction in neutral media microbial fuel cells. Energy Environ. Sci..

[19] Oliot M., Etcheverry L., Mosdale A., Basseguy R., Delia M.-L., Bergel A. (2017). Separator electrode assembly (SEA) with 3-dimensional bioanode and removable air-cathode boosts microbial fuel cell performance. J. Power Sources.

[20] Grattieri M., Suvira M., Hasan K., Minteer S.D. (2017). Halotolerant extremophile bacteria from the Great Salt Lake for recycling pollutants in microbial fuel cells. J. Power Sources.

[21] Grattieri M., Shivel N.D., Sifat I., Bestetti M., Minteer S.D. (2017). Sustainable hypersaline microbial fuel cells: inexpensive recyclable polymer supports for carbon nanotube conductive paint anodes. ChemSusChem.

[22] Schievano A., Colombo A., Grattieri M., Trasatti S.P., Liberale A., Tremolada P., Pino C., Cristiani P. (2017). Floating microbial fuel cells as energy harvesters for signal transmission from natural water bodies. J. Power Sources.

[23] Zhang F., Pant D., Logan B.E. (2011). Long-term performance of activated carbon air cathodes with different diffusion layer porosities in microbial fuel cells. Biosens. Bioelectron..

[24] Sevda S., Dominguez-Benetton X., Vanbroekhoven K., De Wever H., Sreekrishnan T.R., Pant D. (2013). High strength wastewater treatment accompanied by power generation using air cathode microbial fuel cell. Appl. Energy.

[25] Karra U., Muto E., Umaz R., Kölln M., Santoro C., Wang L., Li B. (2014). Performance evaluation of activated carbon-based electrodes with novel power management system for long-term benthic microbial fuel cells. Int. J. Hydrogen Energy.

[26] Dong H., Yu H., Yu H., Gao N., Wang X. (2013). Enhanced performance of activated carbon–polytetrafluoroethylene air-cathode by avoidance of sintering on catalyst layer in microbial fuel cells. J. Power Sources.

[27] Gajda I., Greenman J., Melhuish C., Ieropoulos I. (2015). Simultaneous electricity generation and microbially-assisted electrosynthesis in ceramic MFCs. Bioelectrochemistry.

[28] Antolini E. (2015). Composite materials for polymer electrolyte membrane microbial fuel cells. Biosens. Bioelectron..

[29] Yuan H., Hou Y., Abu-Reesh I.M., Chen J., He Z. (2016). Oxygen reduction reaction catalysts used in microbial fuel cells for energy-efficient wastewater treatment: a review. Mater. Horiz.

[30] Zhang F., Cheng S., Pant D., Van Bogaert G., Logan B.E. (2009). Power generation using an activated carbon and metal mesh cathode in a microbial fuel cell. Electrochem. Commun..

[31] Wei B., Tokash J.C., Chen G., Hickner M.A., Logan B.E. (2012). Development and evaluation of carbon and binder loading in low-cost activated carbon cathodes for air-cathode microbial fuel cells. RSC Adv..

[32] Watson V.J., Delgado C.N., Logan B.E. (2013). Influence of chemical and physical properties of activated carbon powders on oxygen reduction and microbial fuel cell performance. Environ. Sci. Technol..

[33] Watson V.J., Delgado C.N., Logan B.E. (2013). Improvement of activated carbons as oxygen reduction catalysts in neutral solutions by ammonia gas treatment and their performance in microbial fuel cells. J. Power Sources.

[34] Zhang X., He W., Zhang R., Wang Q., Liang P., Huang X., Logan B.E., Fellinger T.-P. (2016). High-performance carbon aerogel air cathodes for microbial fuel cells. ChemSusChem.

[35] Wang H., Wu Z., Plaseied A., Jenkins P., Simpson L., Engtrakul C., Ren Z. (2011). Carbon nanotube modified air-cathodes for electricity production in microbial fuel cells. J. Power Sources.

[36] Ghasemi M., Shahgaldi S., Ismail M., Kim B.H., Yaakob Z., Daud W.R.W. (2011). Activated carbon nanofibers as an alternative cathode catalyst to platinum in a two-chamber microbial fuel cell. Int. J. Hydrogen Energy.

[37] Roustazadeh Sheikhyousefi P., Esfahany M.N., Colombo A., Franzetti A., Trasatti S.P., Cristiani P. (2017). Investigation of different configurations of microbial fuel cells for the treatment of oilfield produced water. Appl. Energy.

[38] Guerrini E., Grattieri M., Faggianelli A., Cristiani P., Trasatti S. (2015). PTFE effect on the electrocatalysis of the oxygen reduction reaction in membraneless microbial fuel cells. Bioelectrochemistry.

[39] Xiao L., Damien J., Luo J., Jang H.D., Huang J., He Z. (2012). Crumpled graphene particles for microbial fuel cell electrodes. J. Power Sources.

[40] Wang Q., Zhang X., Lv R., Chen X., Xue B., Liang P., Huang X. (2016). Binder-free nitrogen-doped graphene catalyst air-cathodes for microbial fuel cells. J. Mater. Chem. A.

[41] Yuan H., He Z. (2015). Graphene-modified electrodes for enhancing the performance of microbial fuel cells. Nanoscale.

[42] Santoro C., Kodali M., Kabir S., Soavi F., Serov A., Atanassov P. (2017). Three-dimensional graphene nanosheets as cathode catalysts in standard and supercapacitive microbial fuel cell. J. Power Sources.

[43] Rossi R., Yang W., Setti L., Logan B.E. (2017). Assessment of a metal-organic framework catalyst in air cathode microbial fuel cells over time with different buffers and solutions. Bioresour. Technol..

[44] Yang W., Logan B.E. (2016). Immobilization of a metal–nitrogen–carbon catalyst on activated carbon with enhanced cathode performance in microbial fuel cells. ChemSusChem.

[45] Zhao F., Harnisch F., Schröder U., Scholz F., Bogdanoff P., Herrmann I. (2006). Challenges and constraints of using oxygen cathodes in microbial fuel cells. Environ. Sci. Technol..

[46] Zhao F., Slade R.C.T., Varcoe J.R. (2009). Techniques for the study and development of microbial fuel cells: an electrochemical perspective. Chem. Soc. Rev..

[47] Kodali M., Santoro C., Serov A., Kabir S., Artyushkova K., Matanovic I., Atanassov P. (2017). Air breathing cathodes for microbial fuel cell using Mn-, Fe-, Co-and Ni-containing platinum group metal-free catalysts. Electrochim. Acta.

[48] Nguyen M.-T., Mecheri B., Iannaci A., D'Epifanio A., Licoccia S. (2016). Iron/Polyindole-based electrocatalysts to enhance oxygen reduction in microbial fuel cells. Electrochim. Acta.

[49] Lu G., Zhu Y., Lu L., Xu K., Wang H., Jin Y., Ren Z.J., Liu Z., Zhang W. (2016). Iron-rich nanoparticle encapsulated, nitrogen doped porous carbon materials as efficient cathode electrocatalyst for microbial fuel cells. J. Power Sources.

[50] Santoro C., Serov A., Gokhale R., Rojas Carbonell S., Stariha S., Gordon J., Artyushkova K., Atanassov P. (2017). A family of Fe-NC oxygen reduction electrocatalysts for microbial fuel cell (MFC) application: relationships between surface chemistry and performances. Appl. Catal., B.

[51] Birry L., Mehta P., Jaouen F., Dodelet J.-P., Guiot S.R., Tartakovsky B. (2011). Application of iron-based cathode catalysts in a microbial fuel cell. Electrochim. Acta.

[52] Iannaci A., Mecheri B., D'Epifanio A., Lazaro Elorri M.J., Licoccia S. (2016). Iron–nitrogen-functionalized carbon as efficient oxygen reduction reaction electrocatalyst in microbial fuel cells. Int. J. Hydrogen Energy.

[53] Nguyen M.-T., Mecheri B., D'Epifanio A., Pepo Sciarria T., Adani F., Licoccia S. (2014). Iron chelates as low-cost and effective electrocatalyst for oxygen reduction reaction in microbial fuel cells. Int. J. Hydrogen Energy.

[54] Costa de Oliveira M.A., Mecheri B., D'Epifanio A., Placidi E., Arciprete F., Valentini F., Perandini A., Valentini V., Licoccia S. (2017). Graphene oxide nanoplatforms to enhance catalytic performance of iron phthalocyanine for oxygen reduction reaction in bioelectrochemical systems. J. Power Sources.

[55] Santoro C., Gokhale R., Mecheri B., D'Epifanio A., Licoccia S., Serov A., Artyushkova K., Atanassov P. (2017). Design of iron (ii) pthalocyanine (FePc) derived oxygen reduction electrocatalysts for high power density microbial fuel cells. ChemSusChem.

[56] Rojas-Carbonell S., Babanova S., Serov A., Artyushkova K., Workman M.J., Santoro C., Mirabal A., Calabrese Barton S., Atanassov P. (2017). Integration of platinum group metal-free catalysts and bilirubin oxidase into a hybrid material for oxygen reduction: interplay of chemistry and morphology. ChemSusChem.

[57] Burkitt R., Whiffen T.R., Yu E.H. (2016). Iron phthalocyanine and MnOx composite catalysts for microbial fuel cell applications. Appl. Catal., B.

[58] Jiang B., Muddemann T., Kunz U., Bormann H., Niedermeiser M., Haupt D., Schlaefer O., Sievers M. (2017). Evaluation of microbial fuel cells with graphite plus MnO_2_ and MoS_2_ paints as oxygen reduction cathode catalyst. J. Electrochem. Soc..

[59] Li X., Hu B., Suib S., Lei Y., Li B. (2010). Manganese dioxide as a new cathode catalyst in microbial fuel cells. J. Power Sources.

[60] Hou Y., Yuan H., Wen Z., Cui S., Guo X., He Z., Chen J. (2016). Nitrogen-doped graphene/CoNi alloy encased within bamboo-like carbon nanotube hybrids as cathode catalysts in microbial fuel cells. J. Power Sources.

[61] Zhao F., Harnisch F., Schroeder U., Scholz F., Bogdanoff P., Herrmann I. (2005). Application of pyrolysed iron (II) phthalocyanine and CoTMPP based oxygen reduction catalysts as cathode materials in microbial fuel cells. Electrochem. Commun..

[62] Kumar R., Singh L., Zularisam A.W., Hai F.I. (2016). Potential of porous Co_3_O_4_ nanorods as cathode catalyst for oxygen reduction reaction in microbial fuel cells. Bioresour. Technol..

[63] Ghasemi M., Ramli Wan Daud W., Rahimnejad M., Rezayi M., Fatemi A., Jafari Y., Somalu M.R., Manzour A. (2013). Copper-phthalocyanine and nickel nanoparticles as novel cathode catalysts in microbial fuel cells. Int. J. Hydrogen Energy.

[64] Yu E.H., Cheng S., Scott K., Logan B.E. (2007). Microbial fuel cell performance with non-Pt cathode catalysts. J. Power Sources.

[65] Huang J., Zhu N., Yang T., Zhang T., Wu P., Dang Z. (2015). Nickel oxide and carbon nanotube composite (NiO/CNT) as a novel cathode non-precious metal catalyst in microbial fuel cells. Biosens. Bioelectron..

[66] Modi A., Singh S., Verma N. (2016). In situ nitrogen-doping of nickel nanoparticle-dispersed carbon nanofiber-based electrodes: its positive effects on the performance of a microbial fuel cell. Electrochim. Acta.

[67] Santoro C., Rojas-Carbonell S., Awais R., Gokhale R., Kodali M., Serov A., Artyushkova K., Atanassov P. (2018). Influence of platinum group metal-free catalyst synthesis on microbial fuel cell performance. J. Power Sources.

[68] Santoro C., Rezaei Talarposhti M., Kodali M., Gokhale R., Serov A., Merino-Jimenez I., Ieropoulos I., Atanassov P. (2017). Microbial desalination cells with efficient platinum-group-metal-free cathode catalysts. ChemElectroChem.

[69] Houghton J., Santoro C., Soavi F., Serov A., Ieropoulos I., Arbizzani C., Atanassov P. (2016). Supercapacitive microbial fuel cell: characterization and analysis for improved charge storage/delivery performance. Bioresour. Technol..

[70] Martinez U., Serov A., Padilla M., Atanassov P. (2014). Mechanistic insight into oxide-promoted palladium catalysts for the electro-oxidation of ethanol. ChemSusChem.

[71] Jia Q., Ramaswamy N., Tylus U., Strickland K., Li J., Serov A., Artyushkova K., Atanassov P., Anibal J., Gumeci C., Barton S.C., Sougrati M.-T., Jaouen F., Halevi B., Mukerjee S. (2016). Spectroscopic insights into the nature of active sites in iron–nitrogen–carbon electrocatalysts for oxygen reduction in acid. NanoEnergy.

[72] Oliot M., Etcheverry L., Bergel A. (2016). Removable air-cathode to overcome cathode biofouling in microbial fuel cells. Bioresour. Technol..

[73] Lacroix R., Da Silva S., Viaplana Gaig M., Rousseau R., Délia M.L., Bergel A. (2014). Modelling potential/current distribution in microbial electrochemical systems shows how the optimal bioanode architecture depends on electrolyte conductivity. Phys. Chem. Chem. Phys..

[74] Oliot M., Galier S., Roux de Balmann E., Bergel A. (2016). Ion transport in microbial fuel cells: key roles, theory and critical review. Appl. Energy.

[75] Erable B., Lacroix R., Etcheverry L., Féron D., Delia M.L., Bergel A. (2013). Marine floating microbial fuel cell involving aerobic biofilm on stainless steel cathodes. Bioresour. Technol..

[76] Weast R.C. (1989). CRC Handbook of Chemistry, and Physics.

[77] Gokhale R., Chen Y., Serov A., Artyushkova K., Atanassov P. (2017). Novel dual templating approach for preparation of highly active Fe-N-C electrocatalyst for oxygen reduction. Electrochim. Acta.

[78] Sebastián D., Serov A., Artyushkova K., Gordon J., Atanassov P., Arico A.S., Baglio V. (2016). High performance and cost-effective direct methanol fuel cells: Fe-N-C methanol-tolerant oxygen reduction reaction catalysts. ChemSusChem.

[79] Rojas-Carbonell S., Artyushkova K., Serov A., Santoro C., Matanovic I., Atanassov P. (2018). Effect of pH on the activity of platinum group metal-free catalysts in oxygen reduction reaction. ACS Catal..

[80] Zhang X., Pant D., Zhang F., Liu J., He W., Logan B.E. (2014). Long-Term performance of chemically and physically modified activated carbons in air cathodes of microbial fuel cells. ChemElectroChem.

[81] Rago L., Cristiani P., Villa F., Zecchin S., Colombo A., Cavalca L., Schievano A. (2017). Influences of dissolved oxygen concentration on biocathodic microbial communities in microbial fuel cells. Bioelectrochemistry.

[82] Santini M., Marzorati S., Fest-Santini S., Trasatti S., Cristiani P. (2017). Carbonate scale deactivating the biocathode in a microbial fuel cell. J. Power Sources.

[83] Daghio M., Gandolfi I., Bestetti G., Franzetti A., Guerrini E., Cristiani P. (2015). Anodic and cathodic microbial communities in single chamber microbial fuel cells. N. Biotech..

[84] Santoro C., Cremins M., Pasaogullari U., Guilizzoni M., Casalegno A., Mackay A., Li B. (2013). Evaluation of water transport and oxygen presence in single chamber microbial fuel cells with carbon-based cathodes. J. Electrochem. Soc..

[85] Torres C.I., Marcus A.K., Rittman B.E. (2008). Proton transport inside the biofilm limits electrical current generation by anode-respiring bacteria. Biotechnol. Bioeng..

[86] Kim B.H., Chang I.S., Gadd G.M. (2007). Challenges in microbial fuel cell development and operation. Appl. Microbiol. Biotechnol..

[87] Santini M., Guilizzoni M., Lorenzi M., Atanassov P., Marsili E., Fest-Santini S., Cristiani P., Santoro C. (2015). Three-dimensional X-ray microcomputed tomography of carbonates and biofilm on operated cathode in single chamber microbial fuel cell. Biointerphases.

[88] Rojas-Carbonell S., Santoro C., Serov A., Atanassov P. (2017). Transition metal-nitrogen-carbon catalysts for oxygen reduction reaction in neutral electrolyte. Electrochem. Commun..

[89] Pasternak G., Greenman J., Ieropoulos I. (2016). Comprehensive study on ceramic membranes for low-cost microbial fuel cells. ChemSusChem.

[90] Yousefi V., Mohebbi-Kalhori D., Samimi A. (2017). Microbial fuel cell (MFC) using commercially available unglazed ceramic wares: low-cost ceramic separators suitable for scale-up. Int. J. Hydrogen Energy.

[91] Rahimnejad M., Bakeri G., Ghasemi M., Zirepour A. (2014). A review on the role of proton exchange membrane on the performance of microbial fuel cell. Polym. Adv. Technol..

[92] Xing Leong J., Ramli Wan Daud W., Ghasemi M., Ben Liew K., Ismail M. (2013). Ion exchange membranes as separators in microbial fuel cells for bioenergy conversion: a comprehensive review. Renew. Sustain. Energy Rev..

[93] Liu H., Logan B.E. (2004). Electricity generation using an air-cathode single chamber microbial fuel cell in the presence and absence of a proton exchange membrane. Environ. Sci. Technol..

[94] Cheng S., Liu H., Logan B.E. (2006). Increased performance of single-chamber microbial fuel cells using an improved cathode structure. Electrochem. Commun..

[95] Luo Y., Zhang F., Wei B., Liu G., Zhang R., Logan B.E. (2011). Power generation using carbon mesh cathodes with different diffusion layers in microbial fuel cells. J. Power Sources.

[96] Zhang X., He W., Yang W., Liu J., Wang Q., Liang P., Huang X., Logan B.E. (2016). Diffusion layer characteristics for increasing the performance of activated carbon air cathodes in microbial fuel cells. Environ. Sci. Water Res. Technol.

[97] Kodali M., Gokhale R., Santoro C., Serov A., Artyushkova K., Atanassov P. (2017). High performance platinum group metal-free cathode catalysts for microbial fuel cell (MFC). J. Electrochem. Soc..

[98] Santoro C., Kodali M., Herrera S., Serov A., Ieropoulos I., Atanassov P. (2018). Power generation in microbial fuel cells using platinum group metal-free cathode catalyst: effect of the catalyst loading on performance and costs. J. Power Sources.

[99] Kodali M., Herrera S., Kabir S., Serov A., Santoro C., Ieropoulos I., Atanassov P. (2018). Enhancement of microbial fuel cell performance by introducing a nano-composite cathode catalyst. Electrochim. Acta.

